# Modeling the effects of treatment adherence challenges on the transmission dynamics of hepatitis C virus

**DOI:** 10.1371/journal.pone.0329543

**Published:** 2025-08-08

**Authors:** Tinashe Victor Mupedza, Laurette Mhlanga, Dennis Mamutse, Mlyashimbi Helikumi, Paride Oresto Lolika, Shingirai Tangakugara Murambiwa, Adquate Mhlanga

**Affiliations:** 1 Department of Mathematics, University of Zimbabwe, Harare, Zimbabwe; 2 Department of Preventive Medicine and Institute for Global Health, Northwestern University, Chicago, Illinois, United States of America; 3 Department of Mathematics and Statistics, College of Science and Technical Education, Mbeya, University of Science and Technology, Mbeya, Tanzania; 4 Department of Mathematics, University of Juba, Central Equatoria, South Sudan; 5 The Program for Experimental and Theoretical Modeling, Division of Hepatology, Department of Medicine, Stritch School of Medicine, Loyola University Chicago, Maywood, Illinois, United States of America; African Population and Health Research Center, KENYA

## Abstract

Infectious disease modeling is crucial for predicting disease progression over time and helps guide decision makers in public health policy. Hepatitis C virus (HCV) prevalence is still increasing in Zimbabwe, a low-middle-income country (LMIC), despite the availability of effective treatments, and the reasons for this increase are not well understood. Our study employed a mathematical model to explain the impact of poor treatment adherence on HCV transmission dynamics in Zimbabwe. We computed the basic reproduction number (${ℛ}_{0}$), a vital metric of disease spread. Equilibrium states of the model were determined, and their stability was investigated. The study demonstrated that an adherence level exceeding 52% causes the reproduction number to drop below 1, curtailing further spread. Our HCV model indicates that variations in re-susceptibility minimally impact outcomes, suggesting that re-susceptibility can often be excluded in such analyses. Our model unraveled the synergistic impact of simultaneously enhancing the recovery rate of acutely infected individuals and treatment adherence on reducing ${ℛ}_{0}$. The study underlines the pressing need for stronger health interventions, including patient education, financial assistance, and rigorous monitoring, to improve treatment adherence. These interventions are paramount in curbing HCV proliferation, particularly in LMICs like Zimbabwe, and can serve as a template for similar settings globally.

## 1 Introduction

Hepatitis C virus (HCV) is a bloodborne pathogen classified under the *Hepacivirus* genus within the *Flaviviridae* family. It can lead to chronic liver diseases such as cirrhosis and hepatocellular carcinoma if left untreated [1,2]. Globally, as of 2019, around 58 million individuals were living with chronic HCV, with 1.5 million new infections occurring annually, including 3.2 million cases among children and adolescents [3]. Despite the availability of highly effective antiviral therapies, only 5% of infected individuals were diagnosed and nearly 0% treated in 2021, according to WHO estimates [4]. This underlines a substantial gap in HCV management and underscores the importance of scaling up testing and treatment, particularly in low- and middle-income countries (LMICs). In Africa, over 90 million cases of hepatitis B and C were reported by 2021, with more than 1% of the population in 18 countries infected with HCV [4]. In Zimbabwe, the burden remains unclear due to poor surveillance systems and sparse epidemiological data. A study by the National Blood Services Zimbabwe (NBSZ) showed an increasing trend in HCV prevalence among blood donors between 2015–2018, with poor treatment adherence highlighted as a contributing factor [5]. Multiple barriers to adherence exist in LMICs, including limited access to affordable medication, stigma, low health literacy, and weak healthcare infrastructure [6,7].

Treatment adherence is critical to prevent drug resistance, improve patient outcomes, and reduce transmission rates [8,9]. Yet, it is often overlooked in both intervention planning and mathematical modeling. While previous modeling studies have explored various aspects of HCV transmission dynamics [10–20], few have integrated the dynamic impact of adherence. Clinical studies such as Bonner *et al*. [21] have discussed its importance, but to our knowledge, no mathematical model has explicitly incorporated adherence as a determinant of HCV transmission dynamics. This study addresses this gap by developing a compartmental model of HCV transmission that explicitly accounts for treatment adherence in Zimbabwe. Our model distinguishes between individuals with chronic infection who are on treatment and those who have discontinued treatment, capturing more nuanced transitions in disease dynamics. By incorporating adherence behavior into the model, we aim to provide actionable insights to inform public health policy and support efforts toward achieving WHO’s 2030 HCV elimination targets [22]. This approach is especially critical for LMICs, where intervention design must consider socio-economic constraints and real-world treatment behaviors.

The subsequent sections of this paper are organized as follows: Sect 2 outlines the model formulation, while Sect 3 computes the reproduction number and conducts a model analysis. Numerical simulations are presented in Sect 4. Sect 5 is the discussion. Sect 6 offers concluding remarks and future directions.

## 2 Model description

The model analyzes the transmission dynamics of HCV within a homogeneously mixing population. The population is categorized into several compartments: susceptible to infection (*S*), acutely infected (*I*), chronically infected under treatment (*C*), chronically infected after quitting treatment prematurely (*C*_*q*_), and recovered individuals (*R*). The total population at time *t*, denoted by *N*, is given by:

$$N=S+I+C+C_{q}+R.$$ (1)

The number of susceptible individuals is augmented by a constant inflow into the population at rate *b*. The parameter *μ* signifies the natural mortality rate applicable to all compartments, *v* represents mortality due to chronic infection under treatment, and ν denotes disease-induced mortality due to chronic infection not under treatment. It is assumed that ν>v, reflecting a higher mortality rate for not under treatment versus under treatment chronic infections. Susceptible individuals acquire the infection at a rate *λ*, defined as:

$$\lambda =\beta_{1}I+\beta_{2}C+\beta_{3}C_{q},$$ (2)

where $\beta_{1},\beta_{2},\beta_{3}$ correspond to the transmission rates from the *I*, *C*, and *C*_*q*_ compartments, respectively. We assumed that $\beta_{2}<\beta_{3}$, since quitting treatment before reaching sustained viral response (SVR) increases HCV viral load, leading to higher transmissibility [23–25]. Recovered individuals (*R*) lose immunity at rate *ϕ*, reverting to the susceptible class (*S*). We introduced the re susceptibility as suggested by [26], where it was stated that reinfection of hepatitis C is a crucial factor to consider in constructing a hepatitis C transmission dynamics model. However, in [26], it had not been included. Acutely infected individuals transition out of the acute stage at rate *r*, while a proportion η of these recover temporarily (*R*), while the remaining (1 − η) develop chronic infection. Those remaining either recover or die. We define θ as the proportion of individuals under treatment who successfully adhere to it. The treatment-related transitions are modeled such that a fraction θ of individuals in the treatment compartment *C* complete treatment successfully, while the remaining fraction 1 − θ fail to adhere and move to the *C*_*q*_ compartment at a rate δ. Thus, the non-adherent individuals transition from *C* to *C*_*q*_ at rate δ(1 − θ). Individuals under treatment recover at rate *ρ*, whereas untreated individuals recover at rate αρ, where α∈(0,1), signifying a slower recovery rate for untreated individuals. Chronically infected individuals on treatment recover faster than those who have quit treatment because the active medication continuously suppresses the viral load and supports the immune system. The compartmental model is governed by the following system of ordinary differential equations (ODEs):

$$\frac{dS}{dt}=b-(\mu +\lambda )S+\phi R,\;\frac{dI}{dt}=\lambda S-(\mu +r)I,\;\frac{dC}{dt}=(1-\eta )rI-\delta (1-\theta )C-(\rho +\mu +v)C,\;\frac{dC_{q}}{dt}=\delta (1-\theta )C-(\alpha \rho +\mu +\nu )C_{q},\;\frac{dR}{dt}=(C+\alpha C_{q})\rho +\eta rI-(\mu +\phi )R.$$ (3)

### Invariant region

Since the HCV model (3) deals with human population which cannot be negative, we need to show that all the solutions of system (3) remain positive with positive initial conditions in the bounded. To guarantee that the system 3 is mathematical well-posed in a feasible region Θ, we establish that:

$$\Theta =\left\{S(t),\;I(t),\;C(t),\;C_{q}(t),\;R(t)\in {\mathbb{R}}_{+}^{5},\;N\leq \frac{b}{\mu }\right\}.$$ (4)

**Theorem 1.** *Let *S*(0)>0,*I*(0)>0,*C*(0)>0,*C**_*q*_*(0)>0,*R*(0)>0 be the initial solutions of the model (*3*); then, *S*(*t*),*I*(*t*),*C*(*t*),*C**_*q*_*(*t*),*R*(*t*) are positive in ${\mathbb{R}}_{+}^{5}$ for all *t*>0.*

Proof of Theorem 1 is presented in Appendix A.

**Theorem 2.** *The region Θ shown in equation (*4*) is bounded in the space ${\mathbb{R}}_{+}^{5}$.*

Proof of Theorem 2 is also provided in Appendix A.

## 3 Reproduction number

The global behavior of our model is largely determined by the basic reproduction number, denoted by ${ℛ}_{0}$. This number represents the average number of secondary infections produced by a single infectious individual when introduced into a completely susceptible population. For the absence of disease, the system exhibits a disease-free equilibrium point ${ℰ}^{0}=(S^{0},A^{0},Q^{0},C^{0},R^{0})$, which is mathematically expressed as:

$${ℰ}^{0}=\left(\frac{b}{\mu },0,0,0,0\right).$$ (5)

To compute the basic reproduction number ${ℛ}_{0}$, we adopt the next-generation matrix method as developed by van den Driessche and Watmough [27]. In accordance with their methodology, we define two matrices *F* and *V* at the disease-free equilibrium. Matrix *F* represents the rate of new HCV infections generated by each infectious class, while matrix *V* incorporates all other transitions between compartments. These matrices are given by:

$$F=(\begin{array}{ccc} \frac{b\beta_{1}}{\mu } & \frac{b\beta_{2}}{\mu } & \frac{b\beta_{3}}{\mu }\\ 0 & 0 & 0\\ 0 & 0 & 0 \end{array}),\;V=(\begin{array}{ccc} m_{1} & 0 & 0\\ -(1-\eta )r & m_{2} & 0\\ 0 & -\delta (1-\theta ) & m_{3} \end{array}).$$ (6)

Subsequently, ${ℛ}_{0}$ is determined as the spectral radius of the next-generation matrix $FV^{-1}$, and can be expressed as:

$${ℛ}_{0}={ℛ}_{I}+{ℛ}_{C}+{ℛ}_{C_{q}}=\frac{b\beta_{1}}{\mu m_{1}}+\frac{br\beta_{2}(1-\eta )}{\mu m_{1}m_{2}}+\frac{rb\delta \beta_{3}(1-\eta )(1-\theta )}{\mu m_{1}m_{2}m_{3}},$$ (7)

where $m_{1}=\mu +r$, $m_{2}=\delta (1-\theta )+\mu +v+\rho$, and $m_{3}=\alpha \rho +\mu +\nu$.

Equation (7) expresses the basic reproduction number *R*_0_ as the sum of three constituent terms:


$$R_{0}=R_{I}+R_{C}+R_{Cq}.$$

- **$R_{I}=\frac{b\beta_{1}}{\mu m_{1}}$**: This term represents the average number of secondary infections generated by an acutely infected individual. The parameter $\beta_{1}$ denotes the transmission rate from the acute class, while $m_{1}=\mu +r$ accounts for the rate at which individuals leave the acute class due to recovery or natural death.
- **$R_{C}=\frac{br\beta_{2}(1-\eta )}{\mu m_{1}m_{2}}$**: This term represents the secondary infections caused by chronically infected individuals who are under treatment. The factor (1−η) reflects the proportion of acutely infected individuals who progress to chronic infection, and $\beta_{2}$ is the transmission rate from the chronic class. The denominator includes $m_{2}=\delta (1\;-\;\theta )\;+\;\mu \;+\;v\;+\;\rho$, which reflects the rates of treatment failure, death, and recovery in the treated class.
- **$R_{Cq}=\frac{rb\delta \beta_{3}(1-\eta )(1-\theta )}{\mu m_{1}m_{2}m_{3}}$**: This component captures the contribution of individuals who initiate but do not complete treatment and transition to the *C*_*q*_ class. The parameter $\beta_{3}$ reflects the higher transmissibility associated with incomplete treatment, and (1−θ) represents the proportion of individuals who fail to adhere. The term $m_{3}=\alpha \rho +\mu +\nu$ includes the reduced recovery rate in this group, as well as disease-induced and natural mortality.

The sub-reproduction numbers ${ℛ}_{I}$, ${ℛ}_{C}$, and ${ℛ}_{C_{q}}$ quantify the contributions to the overall transmission of HCV from individuals in compartments *I*, *C*, and *C*_*q*_, respectively.

If ${ℛ}_{0}<1$, the disease-free equilibrium is locally asymptotically stable. However, under certain conditions, the model may exhibit a backward bifurcation, in which case a stable endemic equilibrium may also exist when ${ℛ}_{0}<1$. Using Theorem 2 in van den Driessche and Watmough (2002) [27], the following result is established.

**Theorem 3.** *The disease-free equilibrium (DFE) of system (3) is locally asymptotically stable (LAS) if *R**_*0*_*<1 and unstable otherwise.*

Local stability offers insights only into the behavior of the disease when the number of HCV infections is close to the equilibrium. If the number of infections is near 0 (albeit not exactly 0), they will decline and converge to 0 over time. But this doesn’t provide any assurance about larger outbreaks. If there is a significant outbreak, the dynamics of HCV might take an unpredictable turn, highlighting the importance of proactive measures even when the disease seems to be under control.

We now investigate the global stability of ${ℰ}^{0}$, the disease-free equilibrium. We claim the following result.

**Theorem 4.** *If ${ℛ}_{0}<1$, the DFE is globally asymptotically stable (GAS) in Θ.*

Proof of Theorem 4 is presented in Appendix B.

Theorem 4 implies that if ${ℛ}_{0}\leq 1$, then the disease free equilibrium is globally asymptotically stable, and the disease will eventually die out, regardless of initial infection levels. However, this relies on the assumption that no other stable equilibria exist when ${ℛ}_{0}\leq 1$, a condition that is examined further in subsequent sections.

**Theorem 5.** *If ${ℛ}_{0}>1$, the disease is uniformly persistent. Specifically, there exists a positive constant ε such that*


$${lim inf}_{t\to \infty }\{I(t)+C(t)+C_{q}(t)\}>\varepsilon$$

*for every solution (*S*,*I*,*C*,*C**_*q*_ *,*R*) of (1) satisfying*


$$I(0)+C(0)+C_{q}(0)>0.$$

Proof of Theorem 5 is presented in Appendix C.

Theorem 5 means that when the reproduction number is greater than 1, (*R*_0_>1), the disease persists in the population. This highlights the importance of of control strategies aimed at reducing *R*_0_ below the critical threshold. The consistent presence of the disease, even at low levels, in the community can act as a reservoir, posing a threat to the wider population. It would be important to keep continuous surveillance and intervention measures at play, within communities especially within high risk groups.

### 3.1 Endemic equilibrium point

Computing and analyzing the endemic equilibrium point (EEP) is key for our study due to its implications on the understanding of the persistence and dynamics of system 3. Through the study of the EEP, we are able to realize valuable insights into system 3 stability, long term behavior and the critical threshold conditions necessary to its sustained existence. The EEP for system 3 is denoted by ${ℰ}^{*}=(S^{*},I^{*},C^{*},C_{q}^{*},R^{*})$. To compute the equilibrium values, we set the right-hand sides of the system (3) to zero:

$$\frac{dS}{dt}=b-(\mu +\lambda )S+\phi R=0,\;\frac{dI}{dt}=\lambda S-(\mu +r)I=0,\;\frac{dC}{dt}=(1-\eta )rI-\delta (1-\theta )C-(\rho +\mu +v)C=0,\;\frac{dC_{q}}{dt}=\delta (1-\theta )C-(\alpha \rho +\mu +\nu )C_{q}=0,\;\frac{dR}{dt}=(C+\alpha C_{q})\rho +\eta rI-(\mu +\phi )R=0,$$ (8)

where the force of infection is given by,

$$\lambda =\beta_{1}I+\beta_{2}C+\beta_{3}C_{q}.$$ (9)

Then, the EEP is given by


$$S^{*}=\frac{m_{1}m_{2}m_{3}}{\beta_{1}m_{2}m_{3}+r(1-\eta )m_{4}}$$

$$I^{*}=\frac{m_{2}m_{3}[(b\beta_{1}-\mu m_{1})m_{2}m_{3}+br(1-\eta )m_{4}](\mu +\phi )}{\begin{array}{c} \left[\beta_{1}m_{2}m_{3}+r(1-\eta )m_{4}][\mu m_{2}m_{3}(\mu +\phi )+rm_{2}(\mu^{2}+(1-\eta )\nu \phi +\mu m_{5}\right)\\ +r\delta (1-\theta )(\mu^{2}+(1-\eta )m_{6}\phi +\mu m_{5})] \end{array}}$$

$$C^{*}=\frac{r(1-\eta )m_{2}[(b\beta_{1}m_{2}m_{3}-\mu (r+\mu )m_{2}m_{3}+br(1-\eta )m_{4}](\mu +\phi )}{\begin{array}{c} \left[\beta_{1}m_{2}m_{3}+r(1-\eta )m_{4}][\mu m_{2}m_{3}(\mu +\phi )+rm_{2}(\mu^{2}+(1-\eta )\nu \phi +\mu m_{5}\right)\\ +r\delta (1-\theta )(\mu^{2}+(1-\eta )m_{6}\phi +\mu m_{5})] \end{array}}$$

$$C_{q}^{*}=\frac{r\delta (1-\eta )(1-\theta )[(b\beta_{1}m_{3}-\mu m_{1}m_{2}m_{3}+br(1-\eta )m_{4}](\mu +\phi )}{\begin{array}{c} \left[\beta_{1}m_{3}+r(1-\eta )m_{4}][\mu m_{2}m_{3}(\mu +\phi )+rm_{2}(\mu^{2}+(1-\eta )\nu \phi +\mu m_{5}\right)\\ +r\delta (1-\theta )(\mu^{2}+(1-\eta )m_{6}\phi +\mu m_{5})] \end{array}}$$

$$R^{*}=\frac{\begin{array}{c} r[(b\beta_{1}-\mu m_{1})m_{2}m_{3}+br(1-\eta )m_{4}][m_{2}(\eta (\mu +\nu )+\rho )\\ +\delta (1-\theta )(\alpha \rho +\eta (\mu +m_{6}))] \end{array}}{\begin{array}{c} \left[\beta_{1}m_{2}m_{3}+r(1-\eta )m_{4}][\mu m_{2}m_{3}(\mu +\phi )+rm_{2}(\mu^{2}+(1-\eta )\nu \phi +\mu m_{5}\right)\\ +r\delta (1-\theta )(\mu^{2}+(1-\eta )m_{6}\phi +\mu m_{5})] \end{array}}$$

where $m_{1}=\mu +r,$ $m_{2}=\mu +\nu +\rho ,$ $m_{3}=\mu +\nu +\rho +\delta (1-\theta ),$ $m_{4}=\beta_{2}(\mu +\nu +\rho )+\beta_{3}\delta (1-\theta ),$ $m_{5}=\nu +\rho +\phi (1-\eta ),$ $m_{6}=\nu +\rho (1-\alpha )$

To examine the local stability of the EEP we will make use of the Centre Manifold Theory, and the next result is established.

**Theorem 6.** *If $\Lambda_{1}>\Lambda_{2}$, *a*>0, then system (*3*) undergoes a backward bifurcation at ${ℛ}_{0}=1$, otherwise *a*<0 and a unique EEP ${ℰ}^{*}$ guaranteed by Theorem 4.1 in [28] is locally asymptotically stable for ${ℛ}_{0}>1$, but close to 1.*

### 3.2 Backward bifurcation analysis

**Fig 1:**
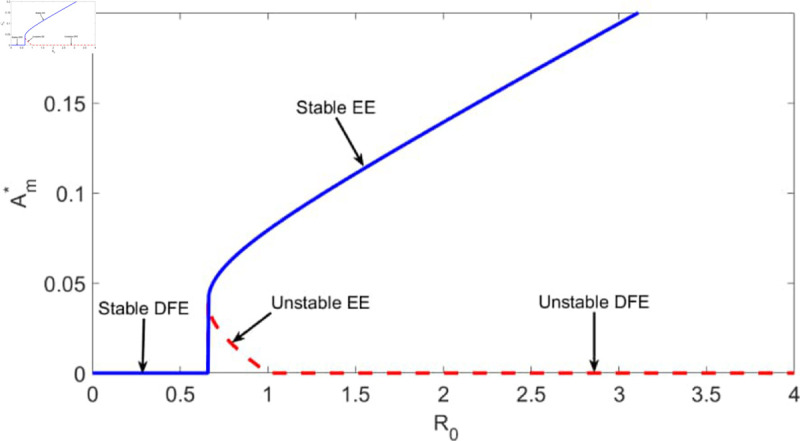
Backward bifurcation diagram for $A^{*}$ of model 3, using the parameter values shown in Table 1. EE denotes endemic equilibrium and DFE denotes disease-free equilibrium.

In this section, we apply center manifold theory to our model (system 3) to establish the conditions under which a backward bifurcation may occur. Specifically, we derive the bifurcation coefficient *a*, following the approach of Castillo-Chavez and Song [28], and demonstrate that under certain parameter conditions, particularly those involving the treatment adherence rate θ, treatment discontinuation rate δ, and the transmission parameters $\beta_{1},\beta_{2},\beta_{3}$; the coefficient *a* becomes positive. This confirms the existence of a backward bifurcation, as presented in Theorem 6. Model system 3 reveals that backward bifurcation arises when $\Lambda_{1}>\Lambda_{2}$ and *a*>0, indicating the coexistence of a stable endemic equilibrium and a stable disease-free equilibrium even when the basic reproduction number satisfies ${ℛ}_{0}<1$, as also stated in Theorem 6. This dynamic is illustrated in Fig 1.

**Table 1:** Model parameters and their interpretations.

Definition	Symbol	Value (Range)	Source
Recruitment rate	*b*	0.01 (0.009-0.06)	[30,31]
Natural mortality rate	μ	0.02 (0.01-0.03)	[30,31]
Disease induced death rate (on treatment)	*v*	0.006 (0.001-0.008)	[32]
Disease induced death rate (not on treatment)	ν	0.055 (0.01-0.065)	Fitted
Disease transmission rate (*I*)	$\beta_{1}$	0.214 (0-3)	[33]
Disease transmission rate (*C*)	$\beta_{2}$	0.016 (0.01-0.02)	Fitted
Disease transmission rate (*C*_*q*_)	$\beta_{3}$	0.05 (0.01-0.1)	Fitted
Re-susceptibility rate	ϕ	0.001 (0.0008-0.0012)	[26]
Acute to recovered rate	*r*	0.163 (0.1-0.18)	Fitted
Recovery rate in acutely infected cases	η	0.3 (0.1-0.4)	[34]
Treatment quitting rate	δ	0.6 (0.4-0.7)	[30]
Treatment adherence proportion	θ	0.5 (0-1)	Assumed
Recovery rate for those with chronic infections	*ρ*	0.053 (0.009-0.0011	Fitted
Slow recovery modification parameter	α	0.001 (0.0009-0.0011)	Fitted

The occurrence of backward bifurcation has some epidemiological implications. Under standard epidemic models, reducing the basic reproduction number ${ℛ}_{0}$ below one is sufficient to eliminate the disease. However, in the presence of a backward bifurcation, this classical threshold rule breaks down: even if ${ℛ}_{0}<1$, the disease may persist due to the coexistence of multiple stable equilibria. This means that conclusions based solely on ${ℛ}_{0}$ can be misleading. From a control perspective, backward bifurcation implies that more aggressive and sustained intervention strategies are necessary not only to drive ${ℛ}_{0}$ below one but also to significantly reduce the number of existing infections. Failing to address the size of the initial infected population may result in persistent endemic transmission, even when standard thresholds are met. Furthermore, the complex dynamics arising from this phenomenon, such as sensitivity to initial conditions complicate eradication efforts. This highlights the critical need to integrate early diagnosis, high treatment coverage, and behavioral interventions into public health strategies for HCV control.

## 4 Numerical simulations

In this section, we perform numerical simulations and sensitivity analyses on our model parameters.

**Fig 2:**
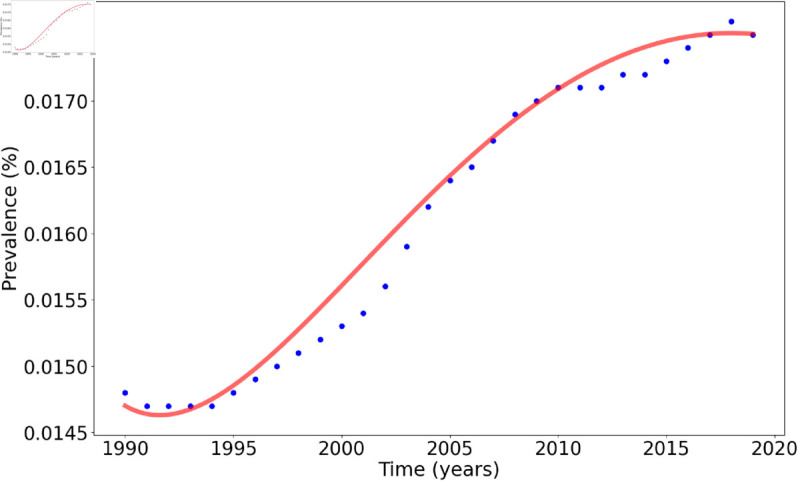
Data fitting results for the HCV dynamics model. The blue markers represent observed data points, while the red line illustrates the model’s predictions. The close alignment between the two shows the model’s accuracy in capturing the underlying dynamics of HCV transmission and progression. The model was fitted to the data from Zimbabwe over a period of 30 years between the years 1990 - 2019, which can be obtained from: https://www.globalhep.org/country-progress/zimbabwe [29], using initial conditions as follows $S_{0}=0.8,\;I_{0}=0.01,\;C_{0}=0.015,\;Cq_{0}=0,\;R_{0}=0$. The root mean square (RMS) was calculated as 0.0001.

The cited parameters, (*b*, *μ*, *v*, $\beta_{1}$, *ϕ*, η, δ), were used with their respective ranges from the literature. By incorporating these cited parameters and their ranges, we ensured that the model remained consistent with established findings and grounded in real-world data. The fitted parameters, including ν (mortality), $\beta_{2}$ and $\beta_{3}$ (transmission rates), *r* (acute to recovered rate), *ρ* (chronic recovery rate), and *α* (slow recovery modification), were determined using Berkeley Madonna’s slider feature to ensure the model closely matched HCV data from Zimbabwe (1990-2019) with an RMS error of 0.0001. The values were kept within biologically plausible ranges based on real-world dynamics, particularly reflecting higher transmission rates for untreated chronic cases and slower recovery rates for individuals not receiving treatment. This fitting process allowed for a model model that accurately represented the progression and transmission of HCV, Fig 2.

**Fig 3:**
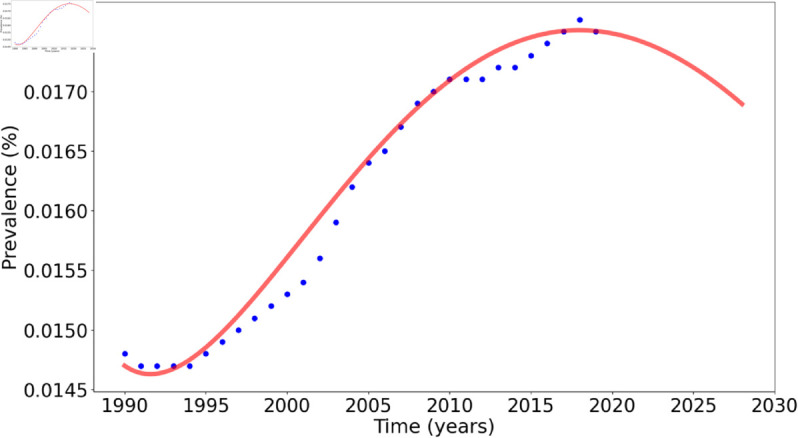
Data fitting results for the HCV dynamics model. The blue markers represent observed data points, while the red line illustrates the model’s predictions. This is an extension of the model fits Fig 3, with projections for the year 2019 to 2028.

The data projection shows that starting from 2019, the HCV cases are declining gradually. Due to the unavailability of data for that period, no verification could be carried out.

**Fig 4:**
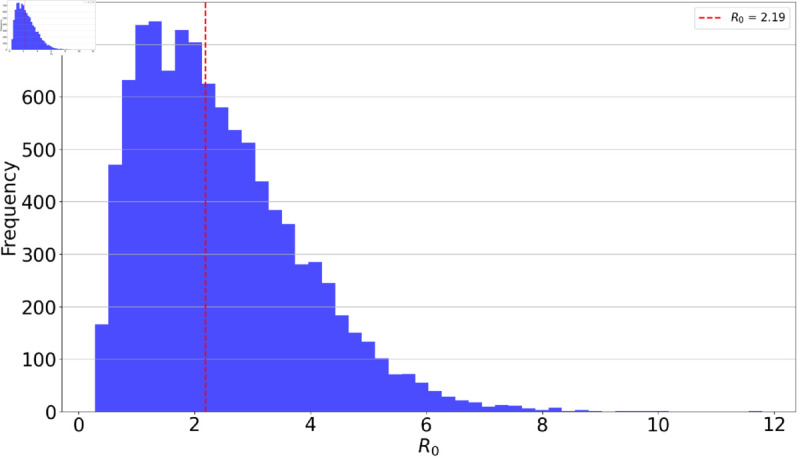
Presents a histogram of the basic reproduction number ${ℛ}_{0}$, computed from 10000 samples using LHS. The distribution highlights the range and frequency of different ${ℛ}_{0}$ values, illustrating their sensitivity to variations in the model parameters listed in Table 1. Notably, the mean value of ${ℛ}_{0}$ across these samples was estimated to be 2.19. The sampling ranges used are as indicated in Table 1.

In Fig 4, the distribution of the basic reproduction number ${ℛ}_{0}$, as determined by LHS, offers notable insights into the variability of the system. While the mean reproduction number stands at 2.19, the spread of the values reveals that certain parameter combinations can lead to either higher or lower ${ℛ}_{0}$ values. The reproduction number that we estimated lies in the same range as the one previously estimated [35]. This variability emphasizes the system’s sensitivity to parameter changes. The broader spread in certain regions of the histogram suggests that even minor alterations in some parameters (as given in Table 1) can have substantial impacts on the system’s dynamics. Understanding this variability is crucial as a higher ${ℛ}_{0}$ indicates a more rapid spread of the infection, necessitating different intervention strategies compared to a scenario with a lower ${ℛ}_{0}$. The results underline the importance of accurate parameter estimation in predicting and controlling the spread of the infection.

**Fig 5:**
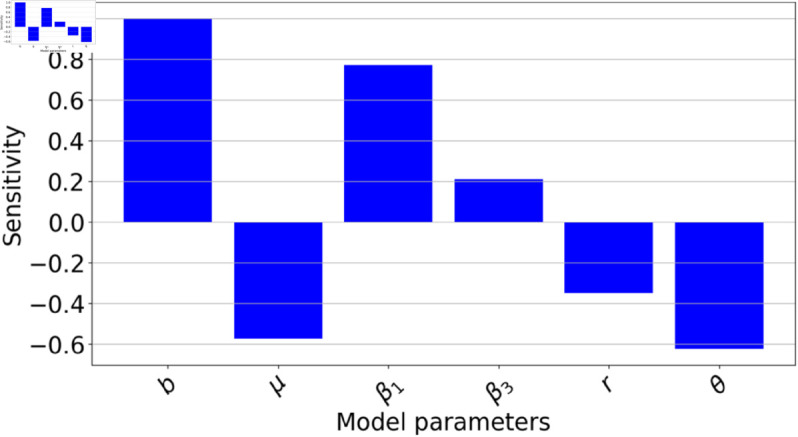
Partial Rank Correlation Coefficient (PRCC) sensitivity analysis results, obtained using LHS with 1000 samples and run in Python version 3.11.0. This highlights the relative influence of model parameters from Table 1 on the basic reproduction number ${ℛ}_{0}$. Parameters with positive PRCCs will increase ${ℛ}_{0}$ when they increase, while those with negative PRCCs will decrease ${ℛ}_{0}$ as they increase. The sampling ranges used are as indicated in Table 1.

**Fig 6:**
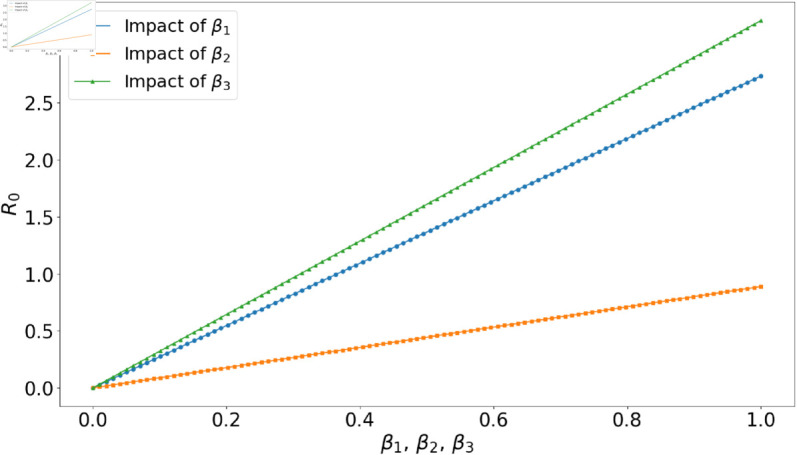
Impact of varying the effective contact rates $\beta_{i},\;i=1,2,3$, on the reproduction number ${ℛ}_{0}$.

In Fig 5, we observed that the recruitment rate exhibits the highest positive index, while the treatment adherence proportion θ presents the most pronounced negative PRCC index. In comparison to Fig 6 where the disease transmission rate for (*C*_*q*_) class $\beta_{3}$ had a greater impact on the reproduction number than the disease transmission rate for (*I*) class $\beta_{1}$, here we found that $\beta_{1}$ exerts a stronger influence than $\beta_{3}$. However, the distinction is not substantial, as both $\beta_{1}$ and $\beta_{3}$ have a more pronounced influence than the disease transmission rate for (*C*) class $\beta_{2}$. Fig 5 illustrates that enhancing treatment adherence can play a pivotal role in reducing the reproduction number. Strategies such as patient education, addressing financial barriers, and consistent follow-ups and monitoring can be essential in curbing HCV spread by decreasing the reproduction number. Furthermore, the recovery rate of those acutely infected *r* is also crucial in this reduction. The parameter *ϕ*, which represents the reinfection rate of those who have recovered, has negligible impact on the reproduction number given its PRCC index is near zero, hence we ddnt include it for the local sensitivity analysis. We further varied *ϕ* to understand the impact of re susceptibility as shown in Fig 12.

**Fig 7:**
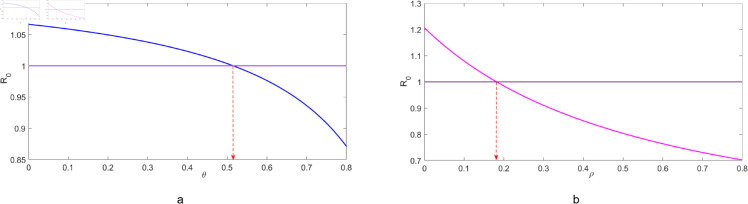
Illustrates the effects of varying: (a) the treatment adherence proportion θ, and (b) the recovery rate for individuals with chronic infections *ρ* on the reproduction number ${ℛ}_{0}$. In this analysis, *ρ* and θ were varied while all other parameters were held constant as displayed in Table 1.

**Fig 8:**
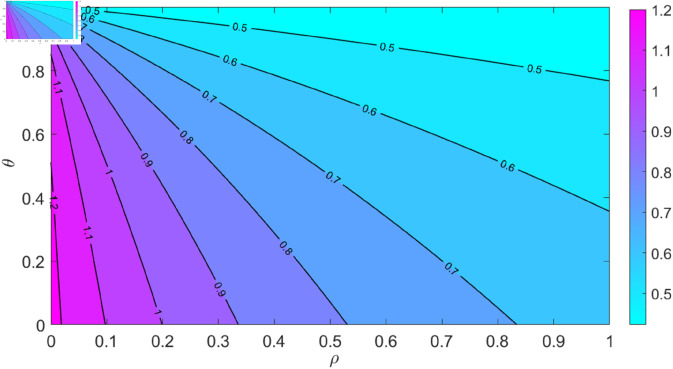
Impact of recovery rate of the chronically infected *ρ* and the proportion adherence θ on the reproduction number ${ℛ}_{0}$. This contour plot shows how *ρ* and θ affect the reproduction number in HCV treatment.

**Fig 9:**
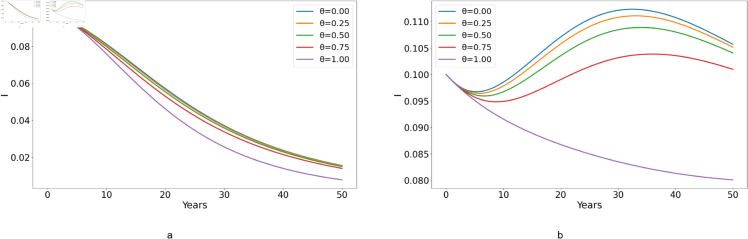
Effect of varying the proportion of individuals adhering to treatment θ on the acutely infected cases I, with (a) ${ℛ}_{0}<1$ and (b) ${ℛ}_{0}>1$.

**Fig 10:**
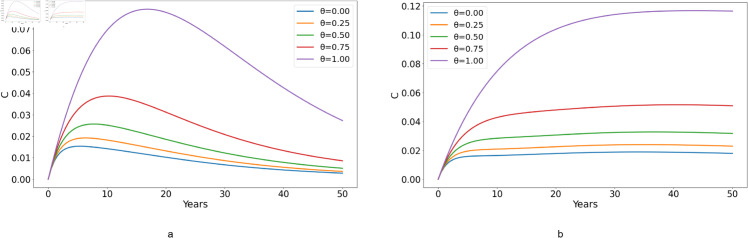
Effect of varying the proportion of individuals adhering to treatment, represented by θ, on the number of chronically infected individuals who are under treatment, denoted by C, with (a) ${ℛ}_{0}<1$ and (b) ${ℛ}_{0}>1$.

**Fig 11:**
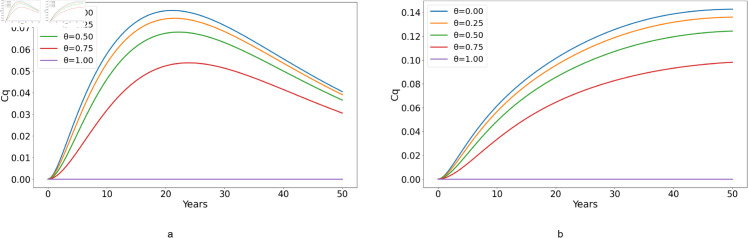
Effect of varying the proportion of individuals adhering to treatment, θ, on the number of chronically infected individuals who are not under treatment, denoted by *C*_*q*_, with (a) ${ℛ}_{0}<1$ and (b) ${ℛ}_{0}>1$.

**Fig 12:**
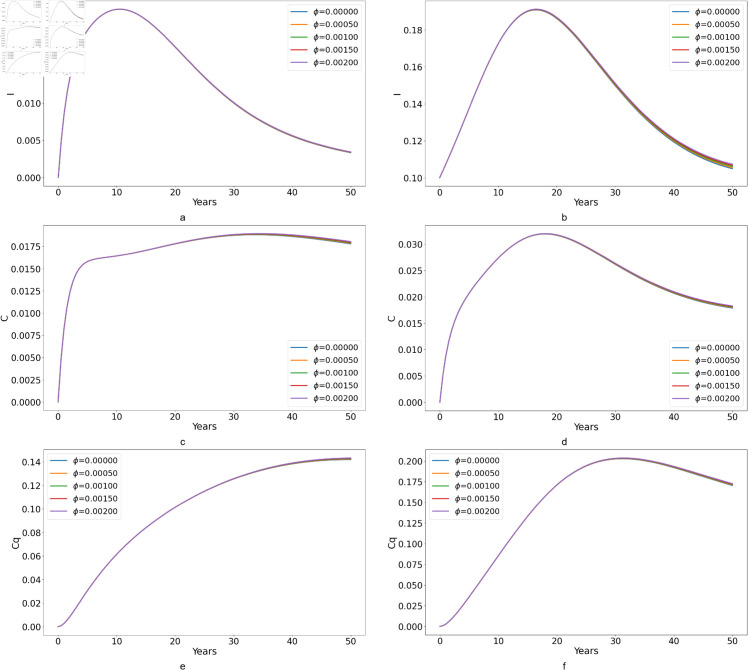
Effect of varying the re-susceptibility rates (*ϕ*) on infection dynamics across varying scenarios of the basic reproduction number (${ℛ}_{0}$). Subplots (*a*) and (*b*) show the number of acutely infected individuals without treatment (*I*) for ${ℛ}_{0}<1$ and ${ℛ}_{0}>1$ respectively. Subplots (*c*) and (*d*) depicts the chronically infected individuals under treatment (*I*) for the corresponding ${ℛ}_{0}$ scenarios. Lastly, subplots (*e*) and (*f*) illustrate the dynamics of chronically infected individuals not under treatment (*C*_*q*_) under ${ℛ}_{0}<1$ and ${ℛ}_{0}>1$ conditions. This figure offers insights into the intricate relationship between treatment adherence and infection outcomes in diverse epidemiological settings.

In Fig 6, we elucidate the consequences of varying $\beta_{1},\;\beta_{2}$, and $\beta_{3}$ values independently, with all other parameters maintained as shown in Table 1. The findings are graphically represented in Fig 6. Most strikingly, $\beta_{3}$ exerts the strongest influence on the reproduction number. This can be attributed to its correlation with the prolonged infectious period experienced by individuals with chronic infections. Parameter $\beta_{1}$, which has more impact over $\beta_{2}$ also plays a significant role, emphasizing its importance in the overall model. In contrast, even though $\beta_{2}$ characterizes the chronically infectious population, its impact is minimized since these individuals are typically under treatment.

Fig 7(*a*) demonstrates that as θ increases, there is a marked decline in ${ℛ}_{0}$. For values of θ<0.5, the decrease is gradual; however, when θ>0.5, the reduction becomes more pronounced. Treatment adherence is particularly effective at reducing the reproduction number once it goes beyond 50% threshold. Additionally, it is worth noting that the reproduction number ${ℛ}_{0}$ goes below 1 when θ>0.52. Fig 7(*b*) illustrates the effect of changing *ρ* on the reproduction number, ${ℛ}_{0}$. Interestingly, the reduction in ${ℛ}_{0}$ appears more consistent across all values of *ρ*. When *ρ* approaches a value of approximately 0.19, we observe that ${ℛ}_{0}$ falls below 1. In essence, as *ρ* increases, there is a systematic reduction in ${ℛ}_{0}$, highlighting the inverse relationship between the two.

Fig 8 provides a comprehensive visualization of the combined effects of varying both *ρ* and θ on the reproduction number. The interplay between these parameters is clear: as θ and *ρ* increase, there’s a corresponding decline in the reproduction number. This indicates that, even with a reasonable recovery rate among the chronically infected, achieving a minimal reproduction number requires exceptionally high adherence rates. Therefore, while the recovery from chronic infection is pivotal, ensuring adherence to treatment protocols becomes equally, if not more, crucial. This highlights the urgency of educational campaigns and the adoption of other proactive strategies that aim to bolster adherence to HCV treatment, to further drive down the reproduction number and, by extension, the spread of the virus.

Fig 9(*a*) reveals that when ${ℛ}_{0}<1$, treatment adherence plays a pivotal role in diminishing the number of *I* cases. Consistently, the number of *I* cases exhibits a decline across the entire adherence spectrum from 0% to 100%. This means that the disease cannot spread in the population because each infected individual, on average, transmits the disease to fewer than one other person. Thus, when ${ℛ}_{0}<1$, the effectiveness of treatment is enhanced by adherence, but the impact is relatively low since the disease is already in decline. Meanwhile, Fig 9(*b*) highlights that at an adherence rate of 75% or below, there’s a surge in the *I* cases. Nonetheless, these start to subside after a span of 30 years. For this case, the impact of adherence is much more effective. When ${ℛ}_{0}>1$, the disease is capable of spreading rapidly through the population, which means that partial adherence is insufficient to control the spread, leading to a substantial increase in infections.

Fig 10(*a*) illustrates the influence of treatment adherence on the C class when ${ℛ}_{0}<1$. Throughout the 0 to 100% range, C cases increase initially but begin to decline after 15 years suggesting that treatment adherence helps control the spread of infection over time, leading to a reduction in new chronic cases eventually. Notably, as the adherence percentages increase, there’s a consistent increase in C cases for ${ℛ}_{0}<1$. This increase in C cases with higher adherence percentages shows the importance of maintaining high adherence to achieve long-term benefits. When comparing Fig 10(*a*) with (*b*), it becomes evident that the C cases count is greater when ${ℛ}_{0}>1$ than when ${ℛ}_{0}<1$, due to more people infected when ${ℛ}_{0}>1$. In Fig 10(*b*), C cases experience an initial rise in the beginning years, subsequently stabilizing for all treatment adherence percentages (0-100%). In this scenario, the initial rise and subsequent stabilization of C cases across all adherence levels suggest that while adherence helps manage the infection, it doesn’t immediately reduce the number of chronically infected individuals due to the higher transmission rate.

Fig 11 illustrates a significant result: at 100% adherence, there are no cases of *C*_*q*_. In Fig 11(*a*), as adherence levels decrease, the number of HCV-infected individuals not under treatment also increases. For all adherence levels, the number of *C*_*q*_ cases increases for up to 20 years before beginning to decline for the remainder of the observation period. In Fig 11(*b*), we observe that *C*_*q*_ cases continue to increase over the entire 50-year period. The impact of adherence is evident; when adherence increases, cases decrease, but the general trend shows an increase. The same explanations from Fig 10 holds.

Comparing Fig 10(b) and Fig 11(b) for *C* (chronic HCV patients adhering to treatment) and *C*_*q*_ (chronic HCV patients who have quit treatment), respectively, we note that individuals adhering to treatment experience an increase in cases that reaches a plateau at around 9 years. This plateau suggests that treatment adherence can stabilize the number of chronic cases, preventing further escalation. In contrast, in cases where individuals do not adhere to treatment, the number of *C*_*q*_ cases continues to increase, indicating a lack of control over the disease progression.

Thus, from Figs 9–11, we can denote that treatment adherence is crucial in the fight against HCV. Ensuring that patients adhere to their prescribed treatment regimens can prevent the increase in chronic cases and help achieve long-term disease control.

We observe that when ${ℛ}_{0}>1$, there is an increase in the number of HCV cases. Additionally, variations in *ϕ* for each transition state appear to have minimal impact on the overall population dynamics. This could be due to the fact that individuals who recover from HCV often develop immunity or have a reduced probability of being re-infected. Furthermore, the rate of re-susceptibility might be considerably lower than the primary infection rate, making it less influential in the disease’s overall spread. Therefore, when constructing mathematical models for HCV, it’s reasonable to omit re-susceptibility, as it doesn’t significantly influence the number of HCV cases.

## 5 Discussion

Mathematical modeling of infectious diseases is pivotal for understanding the spread and control of infectious diseases. In this study, we utilized infectious disease modeling to understand the relationship between treatment adherence and HCV dynamics in Zimbabwe, a LMIC country challenged by this pathogen. Mathematical models have been instrumental in policy formulations, guidance of resource allocation, and helping predict the spread and potential disease outcomes. We developed our mathematical model to reflect the diverse disease stages within the population, providing us with a versatile framework for understanding the various epidemiological transitions that HCV can undergo in this context. We computed the models basic reproduction number (${ℛ}_{0}$), an important epidemiological threshold that measures the disease potential for propagation within the population. We managed to validate our model using data for Zimbabwe HCV cases, from 1990 to 2019. We utilized LHS on 1000 samples to assess the basic reproduction number, ${ℛ}_{0}$. The results indicated that an average ${ℛ}_{0}$ value of 2.19, emphasizing its sensitivity to the parameters detailed in Table 1. Through sensitivity analysis, we were able to pinpoint the pivotal roles played by treatment adherence and the recovery rate of the chronic infected individuals in driving reductions in ${ℛ}_{0}$. We computed the model’s steady states, offering insights into its long-term behavior and disease dynamics. These steady states are crucial for understanding the potential outcomes and stability of the modeled system. We found that variations in *ϕ* have minimal impact on population outcomes, suggesting that re-susceptibility can be reasonably omitted in HCV mathematical models, as recovered individuals often exhibit reduced re-infection rates or develop immunity.

Certainly, the study’s exploration into the effective contact rates, specifically $\beta_{1}$, $\beta_{2}$, $\beta_{3}$ unveils crucial dynamics in HCV transmission. Notably, $\beta_{3}$ emerges as particularly influential, likely due to its association with extended infectious periods characteristic of chronic infections. In contrast, $\beta_{1}$, despite its significant role in disease transmission, also has a pronounced impact, emphasizing its relevance in the disease’s spread. Surprisingly, $\beta_{2}$, which ideally should represent the chronically infectious population, has a more subdued influence, potentially because these individuals are often under treatment. These findings display the intricate interplay of contact rates in HCV transmission and highlight the necessity of understanding and managing these rates to effectively control the disease’s spread. Central to our findings is the relationship between treatment adherence proportion and the reproduction number, ${ℛ}_{0}$. Notably, a pronounced decline in ${ℛ}_{0}$ is evident as adherence rises, particularly beyond the 50% threshold. In fact, the reproduction number drops below the crucial threshold of 1 when adherence surpasses 52%. As adherence levels wane, the population’s pool of chronically infected individuals not under treatment (*C*_*q*_) swells, pointing out the critical importance of adherence promotion strategies in curtailing HCV transmission. This nuanced understanding sheds light on the pressing need for comprehensive interventions aimed at bolstering treatment adherence in LMICs, such as Zimbabwe. Such strategies extend beyond the mere provision of medication to encompass multifaceted initiatives, including patient education, addressing financial barriers to treatment access, and the establishment of rigorous monitoring and follow-up mechanisms. Furthermore, our model unraveled the synergistic impact of simultaneously enhancing the recovery rate of acutely infected individuals and treatment adherence on reducing ${ℛ}_{0}$. This insight suggests that a holistic approach, addressing both the clinical and behavioral aspects of HCV management, can have a profound impact on disease transmission dynamics. Our in-depth sensitivity analysis, implemented through LHS, shed light on the inherent variability within the system. This variability denotes the critical importance of precise parameter estimation in the realm of infectious disease modeling. Minor fluctuations in certain parameters, as detailed in our analysis, can have substantial impacts on the system’s dynamics, thereby emphasizing the need for accurate data collection and model calibration. By fostering a higher rate of recovery among the infected individuals and promoting adherence to treatment regimens, it becomes possible to significantly curtail the virus’s ability to spread within the population.

Our study highlights the minimal impact of re-susceptibility on HCV outcomes, aligning with Wang *et al*.’s suggestion that re-susceptibility may not significantly contribute to transmission dynamics in certain contexts [10]. While Elbasha [12] emphasizes the role of reinfection in HCV transmission, our findings highlight the more substantial influence of increasing recovery rates and treatment adherence in reducing the basic reproduction number, ${ℛ}_{0}$. Both our study and the work of Pitcher *et al*. [14] portray the critical role of treatment adherence in the effort to achieve HCV elimination, with our analysis identifying a pivotal adherence threshold of 52% for bringing ${ℛ}_{0}$ below 1. Furthermore, our conclusions regarding the importance of adherence strategies are consistent with Feng *et al*.’s [15] call for coordinated testing and harm reduction programs to meet WHO elimination goals. While Tatara *et al*. [16] highlight the significance of addressing reinfection risks, our model suggests that re-susceptibility plays a less dominant role, with adherence remaining the key factor for effective control. Additionally, the findings of Mushayabasa and Bhunu [17], which focus on nonadherence among intravenous drug users, complement our study by reinforcing the importance of improving adherence in high-risk populations to reduce HCV transmission.

Our mathematical approach is comprehensive and without limitations. While the most prevalent transmission of HCV occurs among individuals who inject drugs, we did not solely concentrate on this group. Instead, we broadened our perspective to encompass the entire population, utilizing a general transmission model without narrowing our focus. Emphasizing age structures might be crucial for a deeper understanding of the transmission dynamics. Lastly, We cannot say the mathematical is complete, it is still open for further study. Future directions of this model should incorporate age and sex structuring to capture more granular transmission dynamics. This would allow for a more comprehencive understanding of HCV transmission dynamics within specific subpopulations, leading to more tailored intervention strategies. The data used for parameter estimation in this study was obtained from the Global Hepatitis Progress Report for Zimbabwe (https://www.globalhep.org/data-profiles/countries/zimbabwe) [29], which provides modeled trends of hepatitis-related indicators rather than raw data collected annually. While this approach helps to estimate long-term patterns, it introduces inherent limitations that may affect the accuracy of the model’s outputs. One key limitation is that the data points for each year are not the result of direct observation but are instead derived through modeling processes. These modeled estimates are often based on underlying assumptions, extrapolations, and limited available data, which may introduce uncertainty and potential biases in the trends they predict. In resource-constrained settings like Zimbabwe, where accurate and comprehensive surveillance systems may not be in place, these modeled trends can be heavily influenced by the assumptions of the models used to generate them. As a result, the reliability of the data for key parameters such as infection rates, treatment outcomes, and reinfection risks might be reduced. Given that these trends are modeled rather than collected in real time, they may not fully account for rapid epidemiological changes or the immediate impacts of public health interventions. This can introduce imprecision in key estimates and may affect the predictive power of the model. Moving forward, efforts should be made to combine modeled trends with more frequent, real-time data collection to improve the accuracy and robustness of future parameter estimates and predictions.

## 6 Conclusion

Our mathematical model highlights the pivotal role of treatment adherence in shaping the transmission dynamics of HCV in Zimbabwe, a low-middle-income country where the disease remains a major public health concern. The analysis shows that the basic reproduction number, ${ℛ}_{0}$, can fall below the epidemic threshold (${ℛ}_{0}<1$) when adherence levels exceed approximately 52%, provided other key parameters such as the recovery rate of acutely infected individuals are also favorable. Sensitivity analysis revealed that both adherence and recovery rate are among the most influential factors affecting HCV spread, while re-susceptibility has a negligible impact and may be omitted from simplified models. These findings support the implementation of targeted strategies to improve adherence, including patient education, financial support mechanisms, and robust treatment monitoring systems. The model’s responsiveness to changes in parameter values highlights the critical need for accurate parameter estimation in guiding public health decisions. Collectively, this study provides evidence-based insights to inform HCV control efforts through comprehensive and context-specific policy interventions in resource-limited settings.

## Supporting information

Supplementary mathematical proofs Includes detailed proofs of Theorems 1 through 6 related to positivity, boundedness, global and local stability, and backward bifurcation in the model system. (PDF)
