# Supplementary material for: Modeling the effects of treatment adherence challenges on the transmission dynamics of hepatitis C virus
Source: PLoS One. 2025 Aug 8;20(8):e0329543. doi: 10.1371/journal.pone.0329543 (PMC12334037; doi:10.1371/journal.pone.0329543)
Supplement: Supplementary mathematical proofs — (PDF) [file pone.0329543.s001.pdf]

Supplementary Material

[1]Tinashe Victor Mupedza, [2]Laurette Mhlanga, [1]Dennis Mamutse, [3]Mlyashimbi Helikumi,
[4]Paride Oresto Lolika, [1]Shingirai Tangakugara Murambiwa [5]Adequate Mhlanga
[1]*Department of Mathematics, University of Zimbabwe, Box MP 167 Mount Pleasant, Harare, Zimbabwe*
[2]*Department of Preventive Medicine and Institute for Global Health, Northwestern University, Chicago, IL, USA*
[3]*Department of Mathematics and Statistics, College of Science and Technical Education, Mbeya, University of Science and Technology, P.O. Box-131, Mbeya, Tanzania*
[4]*Department of Mathematics, University of Juba, P.O. Box 82 Juba, Central Equatoria, South Sudan*
[5] *The Program for Experimental and Theoretical Modeling, Division of Hepatology, Department of Medicine, Stritch School of Medicine, Loyola University Chicago, Maywood, IL 84101, USA*

Appendix A

Proof of Theorem 1.

Proof. Assume that the initial conditions satisfy

$$S(0) > 0, \quad I(0) > 0, \quad C(0) > 0, \quad C_q(0) > 0, \quad R(0) > 0.$$

Define:

$$\tau = \sup\left\{t > 0 : S(t) > 0,\ I(t) > 0,\ C(t) > 0,\ C_q(t) > 0,\ R(t) > 0\right\}.$$

Since $S(t), I(t), C(t), C_q(t)$, and $R(t)$ are continuous, we deduce that $\tau > 0$.

If $\tau = +\infty$, then positivity holds. However, if $0 < \tau < +\infty$, then at $t = \tau$, we would have:

$$S(\tau) = 0, \quad I(\tau) = 0, \quad C(\tau) = 0, \quad C_q(\tau) = 0, \quad R(\tau) = 0.$$

Now, from the first equation of the model (3), we obtain:

$$\frac{dS}{dt} = b - (\mu + \lambda)S + \phi R.$$

Using the method of integrating factors and performing the necessary calculations, we get:

$$S(\tau) = M_1 S(0) + M_1 \int_0^{\tau} \exp\left(\int (\lambda + \mu) dt\right)(b + \phi R) dt > 0,$$

where

$$M_1 = \exp\left(-(\mu\tau + \int_0^{\tau} \lambda(w) dw)\right) > 0.$$

Since $S(0) > 0$ and $R(0) > 0$, and by the definition of τ, we conclude that $S(\tau) > 0$, contradicting the assumption $S(\tau) = 0$. Hence, $S(\tau) \neq 0$.

Similarly, from the second equation of the model (3), we obtain:

$$\frac{dI}{dt} + (\mu + r)I = \lambda S.$$

Solving, we get:

$$I(\tau) = M_2 I(0) + M_2 \int_0^\tau \exp\left(\int (r+\mu)dt\right) \lambda(w)S(w)dt > 0,$$

where

$$M_2 = \exp\left(-(\mu + r)\tau\right) > 0.$$

Since $I(0) > 0$ and by the definition of τ, it follows that $I(\tau) > 0$, contradicting the assumption $I(\tau) = 0$. Hence, $I(\tau) \neq 0$.

Following the same reasoning, we establish that:

$$C(\tau) > 0, \quad C_q(\tau) > 0, \quad R(\tau) > 0.$$

Thus, by the definition of τ, we conclude that τ is not finite, implying that $\tau = +\infty$. Therefore, all the solutions of the HCV model (3) remain non-negative for all $t > 0$. □

Proof of Theorem 2.

Proof. We obtain the total dynamics of the population by adding the equations of the system (3). This yields

$$N(t) = S(t) + I(t) + C(t) + C_q(t) + R(t).$$

It then follows that

$$\frac{dN}{dt} = b - \mu N - vC - \nu C_q \leq b - \mu N,$$

so that

$$\frac{dN}{dt} + \mu N \leq b. \tag{1}$$

The solution to the first order linear differential inequality (5) is given as:

$$N(t) \leq \frac{b}{\mu}(1 - \exp(-\mu t)) + N(0)\exp(-\mu t). \tag{2}$$

As $t \to \infty$ we obtain $N(t) \leq \frac{b}{\mu}$. So $N(t)$ is a bounded function of time. We can say that Θ is bounded and at limiting equilibrium $\lim_{t\to\infty} N(t) \leq \frac{b}{\mu}$. System (3) is well-posed with all solutions in Θ remaining in Θ or in a neighborhood of Θ if initial conditions are positive. Thus Θ is a positively invariant and is attracting with respect to the flow System (3). □

Appendix B

Proof of Theorem 4

Proof. The proof utilizes the comparison theorem [1], as applied in [2]. All solutions starting in Θ remain in Θ, while all other solutions tend toward Ω. This leads to the conclusion that

$$0 \leq S(t) \leq S^0 \text{ for all } t \geq 0. \tag{3}$$

The three equations for I, C, and C_q in (3) can be formulated as the following differential inequality

$$\begin{pmatrix} \frac{dI}{dt} \\ \frac{dC}{dt} \\ \frac{dC_q}{dt} \end{pmatrix} \leq (F - V) \begin{pmatrix} I \\ C \\ C_q \end{pmatrix}. \tag{4}$$

Considering the given linear ODE system in inequality (13), if the basic reproduction number $R_0 < 1$, then $r(FV^{-1}) < 1$. This denotes that all eigenvalues of $F - V$ lie in the left-half plane [3]. Thus, the system represented by inequality (13) is stable when $R_0 < 1$. Consequently, (I, C, C_q) converges to $(0, 0, 0)$ as $t \to \infty$ for this linear ODE system. By invoking a standard Comparison Theorem (see [1, 4] Theorem B.1; Appendix B), the variables (I, C, C_q) also converge to $(0, 0, 0)$ in the non-linear system described by the three equations in (3). Now, upon examining the first equation of system (3) and substituting $I = C = C_q = 0$ into them, a linear system is derived where S tends to S^0 as $t \to \infty$. Therefore,

$$(S, I, C, C_q, R) \to (S^0, 0, 0, 0, 0) \text{ as } t \to \infty \text{ when } R_0 < 1,$$

which renders $\mathcal{E}^0$ globally asymptotically stable whenever $R_0 < 1$. □

Appendix C

Proof of Theorem 5

Proof. We commence by defining the set X as follows:

$$X = \left\{(S, I, C, C_q, R) \in \mathbb{R}^5_+ : I = C = C_q = 0\right\},$$

which represents all disease-free states of (3). Verification shows that X is positively invariant. Next, we introduce another set $\mathcal{M}$, defined by the intersection of Θ and X:

$$\mathcal{M} = \Theta \cap X.$$

Owing to the positive invariance of both Θ and X, it follows that $\mathcal{M}$ is positively invariant. Importantly, $\mathcal{E}^0$ is an element of $\mathcal{M}$ and attracts all solutions in X, leading to:

$$\Omega(\mathcal{M}) = \{\mathcal{E}^0\}.$$

We further delineate the infected components of (3) by setting:

$$x(t) = \begin{bmatrix} I(t) \\ C(t) \\ C_q(t) \end{bmatrix},$$

which enables us to express the equations as:

$$x'(t) = Y(x)x(t),$$

where the matrix $Y(x)$ is defined as:

$$Y(x) = [F - V].$$

It is evident that $Y(\mathcal{E}^0) = F - V$ and that $Y(\mathcal{E}^0)$ is irreducible. To establish that $\mathcal{M}$ is a uniform weak repeller, we apply Lemma A.4 from [5]. Considering $\mathcal{E}^0$ as a periodic orbit of period $T = 1$ due to its steady-state nature, we identify the fundamental matrix of the solutions for (3) as $P(t, x) = e^{tY}$. Given that the spectral radius of $Y(\mathcal{E}^0) = \mathcal{R}_0 - 1 > 0$, we deduce the spectral radius of $e^{Y(E_0)} > 1$, satisfying condition 2 of Lemma A.4. Upon taking $x = \mathcal{R}_0$, we derive $P(T, \mathcal{R}_0) = e^{Y(\mathcal{R}_0)}$, which is identified as a primitive matrix since $Y(\mathcal{R}_0)$ is irreducible, corroborated by Theorem A.12(i) [6]. This fulfills condition 1 of Lemma A.4, confirming $\mathcal{M}$ as a uniform weak repeller and establishing the weak persistence of the disease. Lastly, we note by definition that $\mathcal{M} = \partial\mathcal{D}$. Being trivially closed, bounded relative to $\mathcal{D}$, and thus compact, $\mathcal{M}$ qualifies as a uniform strong repeller by Theorem 1.3 of [7], which in turn implies the uniform persistence of the disease. □

Appendix D

Proof of Theorem 6

Proof. We use the Centre Manifold Theory to examine the stability the EEP as presented in Theorem 4.1 Chavez and Song in 2004 [8], to ascertain the local asymptotic stability of the endemic equilibrium. We make the following changes on our original variables in order to apply the Centre Manifold Theory, $S = x_1$, $I = x_2$, $C = x_3$, $C_q = x_4$, and $R = x_5$. We now make use of the following vector notation $X = (x_1, x_2, x_3, x_4, x_5)^T$. Thus, model system can now be presented in the form $\frac{dX}{dt} = F = (f_1, f_2, f_3, f_4, f_5)^T$, such that

$$\begin{aligned}
x_1' &= f_1 = b - (\mu + (\beta_1 x_2 + \beta_2 x_3 + \beta_3 x_4))x_1 + \phi x_5,\\
x_2' &= f_2 = (\beta_1 x_2 + \beta_2 x_3 + \beta_3 x_4)x_1 - (\mu + r)x_2,\\
x_3' &= f_3 = (1-\eta)r x_2 - \delta(1-\theta)x_3 - (\rho + \mu + v)x_3,\\
x_4' &= f_4 = \delta(1-\theta)x_3 - (\alpha\rho + \mu + \nu)x_4,\\
x_5' &= f_5 = (x_3 + \alpha x_4)\rho + \eta r x_2 - (\mu + \phi)x_5.
\end{aligned} \tag{5}$$

The method requires us to evaluate the Jacobian of the system (14) at the disease-free equilibrium as denoted by $\mathcal{E}^0$, with $S^0 = x^0$, $I^0 = x_3^0$, $C^0 = x_4^0$, $C_q^0 = x_5^0$ and $R^0 = x_5^0$. Thus,

$$\mathcal{J}(\mathcal{E}^0) = \begin{pmatrix}
-\mu & -\frac{b\beta_1}{\mu} & -\frac{b\beta_2}{\mu} & -\frac{b\beta_3}{\mu} & \phi\\
0 & -m_1 + \frac{b\beta_1}{\mu} & \frac{b\beta_2}{\mu} & \frac{b\beta_3}{\mu} & 0\\
0 & r(1-\eta) & -m_2 & 0 & 0\\
0 & 0 & \delta(1-\theta) & -m_3 & 0\\
0 & r\eta & \rho & \alpha\rho & -\mu-\phi
\end{pmatrix} \tag{6}$$

from which we can establish that

$$\mathcal{R}_0 = \frac{\beta_1 b}{\mu m_1} + \frac{(1-\eta)\beta_2 b r}{\mu m_1 m_2} + \frac{(1-\eta)(1-\theta)\beta_3 \delta b r}{\mu m_1 m_2 m_3}, \tag{7}$$

where

$$S^0 = x^0 = \frac{b}{\mu}, \quad m_1 = \mu + r, \quad m_2 = v + \delta(1-\theta) + \mu + \rho, \quad \text{and} \quad m_3 = \mu + \nu + \alpha\rho.$$

Considering $\beta_3 = \rho_1\beta_1$, $\beta_2 = \rho_0\beta_1$ regardless of whether $\rho_i \in (0,1)$ or $\rho_i \geq 1$ for $i = 0$ and 1. Considering the case for $\mathcal{R}_0 = 1$ and taking β_1 as our bifurcation parameter and solving for β_1, we obtain

$$\beta^* = \beta_1 = \frac{\mu m_1 m_2 m_3}{b\{m_2 m_3 + r(1-\eta)[\rho_0 m_3 + \delta(1-\theta)\rho_1]\}}.$$

It is worth noting that the linearized system of our transformed equation with the bifurcation point β^* comprises of a simple eigenvalue. Therefore, we can apply the center manifold theory to analyze the dynamics of the system near $\beta^* = \beta_1$. It can be demonstrated that the Jacobian of our model system has a right

eigenvalue related with the following zero eigenvalue $u = (u_1, u_2, u_3, u_4, u_5)^T$, where

$$\begin{cases} u_1 = \dfrac{\phi u_5}{\mu} - \dfrac{m_1 m_2 u_3}{\mu r(1-\eta)}, \quad u_2 = \dfrac{m_2 u_3}{r(1-\eta)} > 0, \quad u_3 > 0, \\ u_4 = \dfrac{\delta(1-\theta)u_3}{m_3} > 0, \quad u_5 = \dfrac{\eta m_2 u_3}{(1-\eta)(\mu+\phi)} + \dfrac{\rho u_3}{\mu+\phi} + \dfrac{\alpha\rho\delta(1-\theta)u_3}{(\mu+\phi)m_3} > 0. \end{cases} \tag{8}$$

The left eigenvector of $\mathcal{J}(\mathcal{E}^0)$ associated with the zero eigenvalue at $\beta^* = \beta_1$ is given by

$$v = (v_1, v_2, v_3, v_4, v_5)^T,$$

where

$$\begin{cases} v_1 = 0, \quad v_2 > 0, \quad v_3 = \dfrac{b[m_3\beta_2 + \delta(1-\theta)\beta_3]v_2}{\mu m_2 m_3} > 0, \\ v_4 = \dfrac{b\beta_3 v_2}{\mu m_3} > 0, \quad v_5 = 0. \end{cases} \tag{9}$$

We then apply Theorem 4.1 from [8].

Computation of the bifurcation parameters a and b

For model system (14), the respective non-zero partial derivatives of F at the infection-free equilibrium are given by:

$$\begin{aligned} \frac{\partial^2 f_1}{\partial x_1 \partial x_2} &= \frac{\partial^2 f_1}{\partial x_2 \partial x_1} = -\beta_1, \quad \frac{\partial^2 f_1}{\partial x_1 \partial x_3} = \frac{\partial^2 f_1}{\partial x_3 \partial x_1} = -\beta_2, \quad \frac{\partial^2 f_1}{\partial x_1 \partial x_4} = \frac{\partial^2 f_1}{\partial x_4 \partial x_1} = -\beta_3, \\ \frac{\partial^2 f_2}{\partial x_1 \partial x_2} &= \frac{\partial^2 f_2}{\partial x_2 \partial x_1} = \beta_1, \quad \frac{\partial^2 f_2}{\partial x_1 \partial x_3} = \frac{\partial^2 f_2}{\partial x_3 \partial x_1} = \beta_2, \quad \frac{\partial^2 f_2}{\partial x_1 \partial x_4} = \frac{\partial^2 f_2}{\partial x_4 \partial x_1} = \beta_3, \end{aligned} \tag{10}$$

From (15) we have that

$$a = 2u_1 v_2(u_2\beta_1 + u_3\beta_2 + u_4\beta_3). \tag{11}$$

Since the sign of u_1 is unknown, we introduce two auxiliary variables, $\Lambda_1 = \dfrac{\phi u_5}{\mu}$ and $\Lambda_2 = \dfrac{m_1 m_2 u_3}{\mu r(1-\eta)}$. Thus, we have $u_1 = \Lambda_1 - \Lambda_2$. Consequently, u_1 is positive when $\Lambda_1 > \Lambda_2$ and negative when $\Lambda_1 < \Lambda_2$. Therefore, the sign of a (whether it is positive or negative) depends on whether $\Lambda_1 > \Lambda_2$ or $\Lambda_1 < \Lambda_2$.

For the sign of b, it is associated with the following non-vanishing partial derivatives of F,

$$\begin{aligned} \frac{\partial^2 f_1}{\partial x_2 \partial \beta_1} &= -\frac{b}{\mu}, \quad \frac{\partial^2 f_1}{\partial x_3 \partial \beta_1} = -\frac{\rho_0 b}{\mu}, \quad \frac{\partial^2 f_1}{\partial x_4 \partial \beta_1} = -\frac{\rho_1 b}{\mu}, \\ \frac{\partial^2 f_2}{\partial x_2 \partial \beta_1} &= \frac{b}{\mu}, \quad \frac{\partial^2 f_2}{\partial x_3 \partial \beta_1} = \frac{\rho_0 b}{\mu}, \quad \frac{\partial^2 f_2}{\partial x_4 \partial \beta_1} = \frac{\rho_1 b}{\mu}. \end{aligned} \tag{12}$$

From the expressions in (21), it follows that

$$b = \frac{b}{\mu} v_2(u_2 + \rho_0 u_3 + \rho_1 u_4) > 0, \tag{13}$$

Hence, we establish that $b > 0$. The sign of a is determined by the relationship between Λ_1 and Λ_2, where $a > 0$ if $\Lambda_1 > \Lambda_2$ and $a < 0$ if $\Lambda_1 < \Lambda_2$. The positivity of b is a common feature in epidemic models, often influenced by the choice of φ (denoted as ϕ in [8]) as a bifurcation parameter, ensuring $b > 0$. Applying Theorem 4.1 from [8], particularly items (i) and (iv), we conclude that when $a > 0$ and $b > 0$, system (3) undergoes a backward bifurcation at $\mathcal{R}_0 = 1$. Conversely, when $a < 0$, Theorem 4.1 in [8] guarantees the existence of a unique endemic equilibrium $\mathcal{E}^*$, which is locally asymptotically stable for $\mathcal{R}_0 > 1$ but sufficiently close to 1.

Thus, by employing Theorem 4.1 from [8], specifically items (i) and (iv), we can validate Theorem 6.

□

References

[1] Lakshmikantham V, Leela S, Martynyuk AA. *Stability analysis of nonlinear systems.* New York: Marcel Dekker, Inc.; 1989.

[2] Mhlanga A, Bhunu CP, Mushayabasa S. HSV-2 and substance abuse amongst adolescents: insights through mathematical modelling. *Journal of Applied Mathematics.* 2014;2014:104819.

[3] Van den Driessche P, Watmough J. Reproduction number and sub-threshold endemic equilibria for compartmental models of disease transmission. *Mathematical Biosciences.* 2002;180:29–48.

[4] Smith HL, Waltman P. *The theory of the chemostat.* Cambridge: Cambridge University Press; 1995.

[5] Ackleh AS, Maa B, Salceanu PL. Persistence and global stability in a selection-mutation size-structured model. *Journal of Biological Dynamics.* 2011;5:436–453.

[6] Smith HL, Waltman P. *The theory of the chemostat.* Cambridge: Cambridge University Press; 1995.

[7] Thieme HR. Persistence under relaxed point-dissipativity. *SIAM Journal on Mathematical Analysis.* 1993;24:407–435.

[8] Castillo-Chavez C, Song B. Dynamical models of tuberculosis and their applications. *Mathematical Biosciences and Engineering.* 2004;1:361–404.
